# The complete mitochondrial genome of *Phthiridium szechuanum* (Nycteribiidae, Diptera)

**DOI:** 10.1080/23802359.2023.2171245

**Published:** 2023-02-05

**Authors:** Xianzheng Zhang, Xiaobin Huang, Yujuan Wang, Huijuan Yang, Jinting Yang, Xiaoyan Zheng

**Affiliations:** a Institute of Pathogens and Vectors, Yunnan Provincial Key Laboratory for Zoonosis Control and Prevention, Dali University, Dali, China; b Jilin Provincial Key Laboratory of Animal Resource Conservation and Utilization, Northeast Normal University, Changchun, China

**Keywords:** Mitochondrial genome, Nycteribiidae, *Phthiridium szechuanum*, phylogeny

## Abstract

*Phthiridium szechuanum* is a bat surface parasite under the family Nycteribiidae that prefers to roost in the hair of bats to feed on their blood. In this study, the complete mitochondrial genome of *P. szechuanum* was studied for the first time using Illumina sequencing technology. The mitochondrial genome was 14,896 bp in size and was predicted to encode 37 genes including 13 protein-coding genes, 22 transfer RNA genes, and 2 ribosomal RNA genes. Phylogenetic trees were constructed using the IQ-TREE web server and phylogenetic analysis was performed using the maximum likelihood method, and *P. szechuanum* was found to be phylogenetically closest to *Basilia ansifera*. These data will provide a molecular biological approach to the species identification of *P. szechuanum* and provide a new reference for further studies on the population genetics and phylogeny of the family Nycteribiidae.

## Introduction

The family Nycteribiidae is a class of obligate bat ectoparasites under the order Diptera and Hippoboscoidea, which together with the family Streblidae are known as bat flies (Porter et al. 2022). Members of the family Nycteribiidae have no wings, flat chests, small or missing eyes, and look like spiders. They like to live in caves inhabited by bats and feed on the blood of bats (Staegemann 2016). Previous studies have demonstrated that spider flies are important vectors for a variety of pathogens (Morse et al. 2012; Obame-Nkoghe et al. 2016). Nycteribiidae contains three subfamilies (Archinycteribiinae, Nycteribiinae, Cyclopodiinae), 11 genera, and 275 species, of which about 70% are distributed in the Western Hemisphere (Graciolli et al. 2007).

*Phthiridium szechuanum* (Theodor and Moscona 1954) is a species of the family Nycteribiidae, subfamily Nycteribiinae, genus Phthiridium. It is small (generally about 2 mm long) and usually lives on the fur on the back and abdomen of bats (Theodor 1967). At present, due to the lack of research on its genetic information, the phylogenetic research of *P. szechuanum* is not clear enough. In this study, we reported the complete mitogenome of *P. szechuanum* for the first time, this will help resolve outstanding questions related to the systematics and evolution of bat flies, and provide a better understanding of the phylogenetic relationships in the order of Diptera.

## Materials and methods

### Sample collection

**Figure 1.**
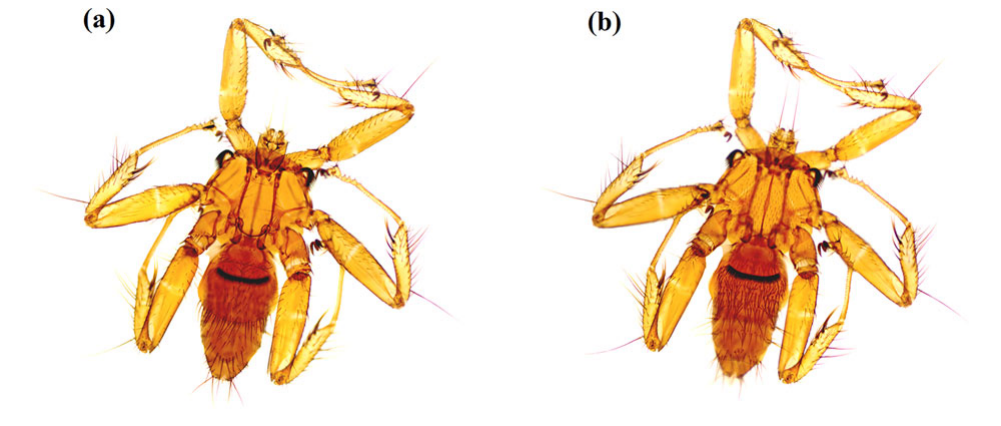
Due to the small size of the specimen, a stereomicroscope (Moticam 2506, China) was used to photograph *P. szechuanum* (♀, (a) is the back side, (b) is the ventral side).

The specimens of *P. szechuanum* were collected in the Bat Cave (25°37′28ʺN, 103°33′54ʺE) in Zhanyi District, Qujing City, Yunnan Province. It was taken from the body surface of *Rhinolophus sinicus*, nine specimens were collected in total, and one specimen was selected as a voucher (Figure 1) and preserved in the Institute of Pathogens and Vector Biology of Dali University, Dali, China (https://www.dali.edu.cn/kxyj/yjs/1611.htm, voucher number: DLQJ2022001 Contact person: Xiaobin Huang, huangxb633@nenu.edu.cn), and the other eight were used for DNA extraction. Species identification was performed by Xianzheng Zhang based on morphological features using a Leica DM3000 LED biological microscope (Germany) according to the identification keys provided by Oskar Theodor (Theodor 1967). The sample was preserved in 95% ethanol and stored at −20 °C until utilization.

Statement: All methods and procedures for capturing small mammals were performed by the guidelines and regulations approved by the Animal Ethics Committee of Dali University under approval number MECDU-202104-27.

### DNA extraction, mitochondrial genome sequencing, and annotation

Firstly, the genomic DNA of *P. szechuanum* was extracted using DNeasy Blood and Tissue Kit (QIAGEN). Then, Sequencing of the complete mitochondrial genome on the Illumina Novoseq 6000 platform and used MitoZ 2.3 (https://doi.org/10.1093/nar/gkz173) to assemble the mitochondrial genome ((Meng et al. 2019). The complete mitochondrial genome sequence was annotated by MITOS and combined with GeSeq (Tillich et al. 2017) to predict the genomès coding genes, tRNA, and rRNA (Bernt et al. 2013). Finally, the mitochondrial genome cycle graph of *P. szechuanum* were mapped using OGDRAW (Greiner et al. 2019).

### Sequence analysis

Geneious Prime was used to calculate the nucleobase composition of *P. szechuanum. Carcinoscorpius rotundicauda* JX437074 (Sarmiento et al. 2021) and *Limulus Polyphemus* JX983598 (Simpson et al. 2017) were used as outgroups, based on 13 mitochondrial protein-coding gene sequences, Mega X software (Kumar et al. 2018) was chosen to perform multiple sequence alignment of these sequences and a phylogenetic tree between *P. szechuanum* and 11 other species was constructed using the IQ-TREE web server (Trifinopoulos et al. 2016) using the maximum-likelihood method. Clade support was assessed using a nonparametric bootstrap method with 1000 replicates. The GenBank accession numbers of these 11 species are listed below. *Sarcophaga albiceps* KT444443 (Liao et al. 2016), *Sarcophaga impatiens* JN859549 (Nelson et al. 2012), *Gasterophilus intestinalis* KU236025 (Gao et al. 2016), *Calliphora vicina* JX913760 (Nelson et al. 2012), *Calliphora vomitoria* KT444440 (Ren et al. 2016), *Ectophasia rotundiventris* MK644821 (Li et al. 2017), *Elodia flavipalpis* JQ348961 (Zhao et al. 2013), *Basilia ansifera* MZ826150 (Porter et al. 2022), *Dipseliopoda setosa* MZ826151 (Porter et al. 2022), *Paradyschiria parvula* MK896865 (Trevisan et al. 2019), *Paratrichobius longicrus* MK896866 (Trevisan et al. 2019).

## Results and discussion

### Mitogenomic organization and nucleotide composition

**Figure 2.**
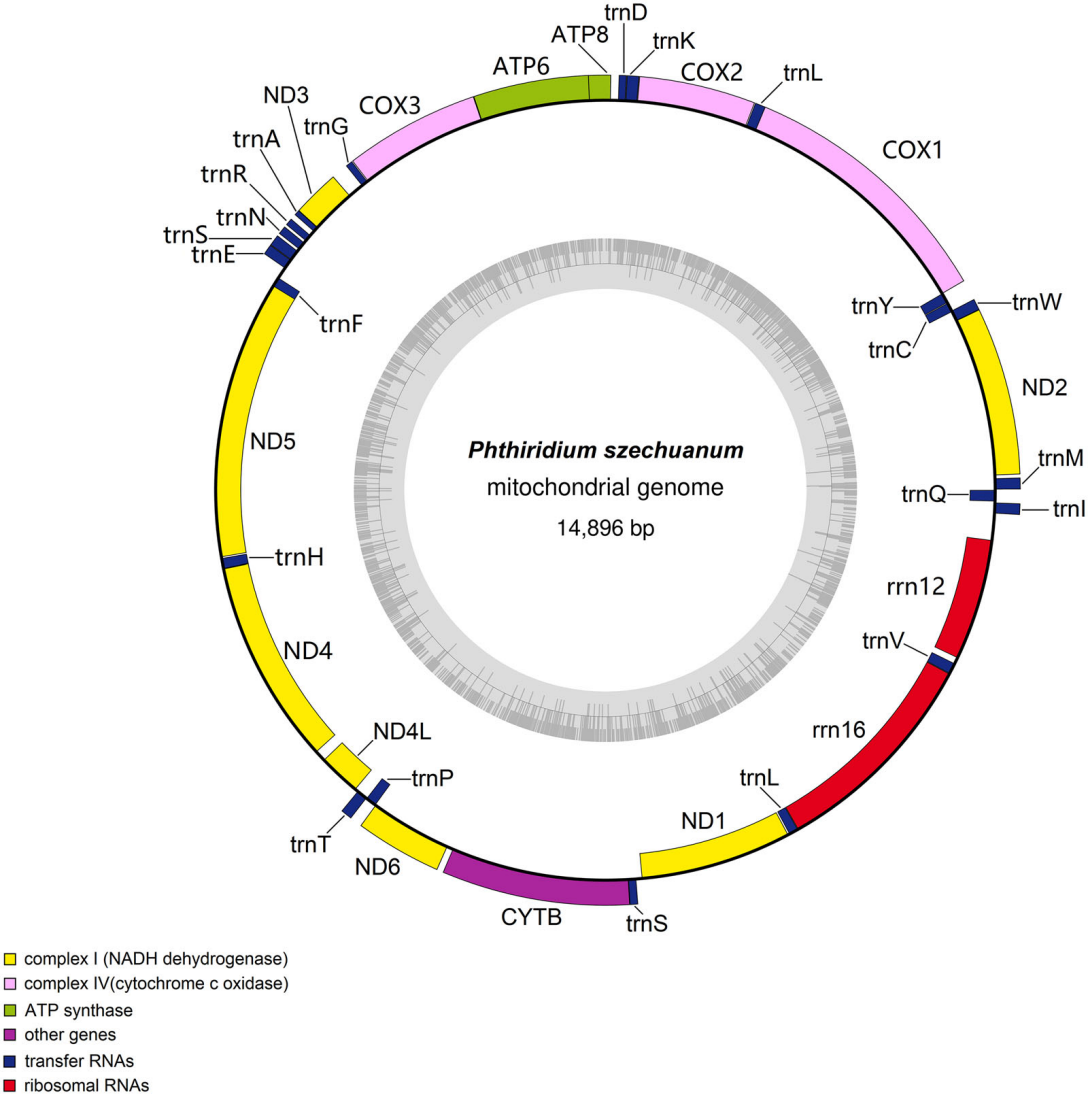
The genome cycle graph of *P. szechuanum.*

The complete mitochondrial genome of *P. szechuanum* (GenBank accession number: OP459298) is 14,896 bp (Figure 2), which contains 37 genes (13 protein-coding genes (PCGs), 22 tRNA genes, two rRNA genes (12S and 16S), and one control region). The gene structure of *P. szechuanum* is similar to previous dipteran mitochondrial genome studies (Zhou et al. 2017). The nucleotide composition of *P. szechuanum* is 39.8% of A, 43.4% of T, 6% of G, and 10.8% of C. Among them, the proportion of A + T (83.2%) is higher than that of G + C (16.8%).

### Protein-coding genes

Among the 13 PCGs, Five PCGs were started with ATG codon; *atp6* and *nad3* were started with ATA; *atp8*, *nad2*, *nad6*, and *cytb* were started with ATT; *cox1* using TCG as a start codon, and *nad5* was initialed by TTG codon. Six PCGs were terminated with TAA stop codon; while *nad1*, *nad3*, and *cytb* genes were stopped at TAG codon; *cox2* and *nad4* were ended with the incomplete stop codon TA; *cox1* and *nad5* were ended at single T.

### Phylogenetic analysis

**Figure 3.**
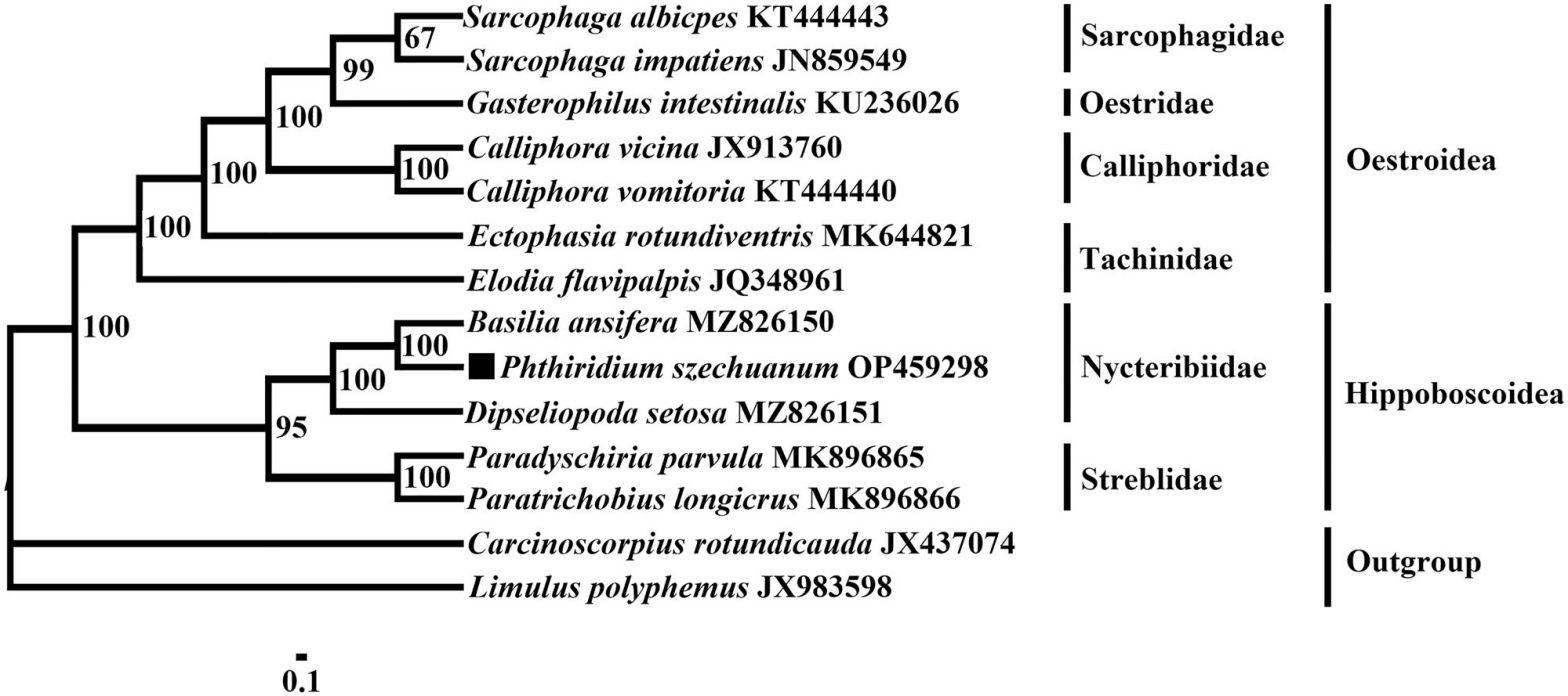
The phylogenetic relationships of *P. szechuanum* and 11 Diptera species were inferred from maximum-likelihood analysis based on 13 PCGs. Numbers on branches represent bootstrap values and square indicates the data sequenced in this study.

As shown in Figure 3, within the Phylogenetic tree, the clustering results of each branch are consistent with those of the taxonomy. *P. szechuanum* was clustered together with species in the family Nycteribiidae and shared a close relationship with *B. ansifera*, supporting *P. szechuanum* among the family Nycteribiidae.

## Conclusions

In this study, we reported the first complete mitochondrial genome sequencing of *P. szechuanum*. The mitochondrial genome sequence will be an important resource for further molecular studies with *P. szechuanum*, it can be used as a novel reference for further studies on the family Nycteribiidae.

## Data Availability

The genome sequence data that support the findings of this study are openly available in GenBank of NCBI at (https://www.ncbi.nlm.nih.gov/) under accession nos. OP459298. The associated Bio Project, BioSample, and SRA experiment numbers are PRJNA883607, SAMN30974214, and SRR21749916, respectively.

## References

[CIT0001] Bernt M, Donath A, Jühling F, Externbrink F, Florentz C, Fritzsch G, Pütz J, Middendorf M, Stadler PF. 2013. MITOS: improved *de novo* metazoan mitochondrial genome annotation. Mol Phylogenet Evol. 69(2):313–319. 22982435 10.1016/j.ympev.2012.08.023

[CIT0002] Gao DZ, Liu GH, Song HQ, Wang GL, Wang CR, Zhu XQ. 2016. The complete mitochondrial genome of *Gasterophilus intestinalis*, the first representative of the family Gasterophilidae. Parasitol Res. 115(7):2573–2579. 26987644 10.1007/s00436-016-5002-9

[CIT0003] Graciolli G, Autino AG, Claps GL. 2007. Catalogue of American Nycteribiidae (Diptera, Hippoboscoidea). Rev Bras Entomol. 51(2):142–159.

[CIT0004] Greiner S, Lehwark P, Bock R. 2019. OrganellarGenomeDRAW (OGDRAW) version 1.3. 1: expanded toolkit for the graphical visualization of organellar genomes. Nucleic Acids Res. 47(W1):W59–W64. 30949694 10.1093/nar/gkz238 PMC6602502

[CIT0005] Kumar S, Stecher G, Li M, Knyaz C, Tamura K. 2018. MEGA X: molecular evolutionary genetics analysis across computing platforms. Mol Biol Evol. 35(6):1547–1549. 29722887 10.1093/molbev/msy096 PMC5967553

[CIT0006] Li X, Ding S, Hou P, Liu X, Zhang C, Yang D. 2017. Mitochondrial genome analysis of *Ectophasia roundiventris* (Diptera, Tachinidae). Mitochondrial DNA Part B. 2(2):457–458. 33490457 10.1080/23802359.2017.1357447 PMC7800338

[CIT0007] Liao H, Yang X, Li Z, Ding Y, Guo Y. 2016. The complete mitochondria genome of *Parasarcophaga albiceps* (Diptera: sarcophagidae). Mitochondrial DNA Part A. 27(6):4696–4698. 10.3109/19401736.2015.1106507 26642375

[CIT0008] Meng G, Li Y, Yang C, Liu S. 2019. MitoZ: a toolkit for animal mitochondrial genome assembly, annotation and visualization. Nucleic Acids Res. 47(11):e63–e63. 30864657 10.1093/nar/gkz173 PMC6582343

[CIT0009] Morse SF, Olival KJ, Kosoy M, Billeter S, Patterson BD, Dick CW, Dittmar K. 2012. Global distribution and genetic diversity of Bartonella in bat flies (Hippoboscoidea, Streblidae, Nycteribiidae). Infect Genet Evol. 12(8):1717–1723. 22771358 10.1016/j.meegid.2012.06.009

[CIT0010] Nelson LA, Lambkin CL, Batterham P, Wallman JF, Dowton M, Whiting MF, Yeates DK, Cameron SL. 2012. Beyond barcoding: a mitochondrial genomics approach to molecular phylogenetics and diagnostics of blowflies (Diptera: Calliphoridae). Gene. 511(2):131–142. 23043935 10.1016/j.gene.2012.09.103

[CIT0011] Nelson LA, Cameron SL, Yeates DK. 2012. The complete mitochondrial genome of the flesh fly, Sarcophaga impatiens Walker (Diptera: Sarcophagidae). Mitochondrial DNA. 23(1):42–43. 22292894 10.3109/19401736.2011.644042

[CIT0012] Obame-Nkoghe J, Rahola N, Bourgarel M, Yangari P, Prugnolle F, Maganga GD, Leroy E-M, Fontenille D, Ayala D, Paupy C. 2016. Bat flies (Diptera: Nycteribiidae and Streblidae) infesting cave-dwelling bats in Gabon: diversity, dynamics and potential role in *Polychromophilus melanipherus* transmission. Parasit Vectors. 9(1):1–12. 27286888 10.1186/s13071-016-1625-z PMC4902993

[CIT0013] Porter ML, Lutz H, Steck M, Chong RA. 2022. The complete mitochondrial genomes and phylogenetic analysis of two Nycteribiidae bat flies (Diptera: Hippoboscoidea). Mitochondrial DNA Part B. 7(8):1486–1488. 35989876 10.1080/23802359.2022.2107450 PMC9387315

[CIT0014] Ren L, Guo Q, Yan W, Guo Y, Ding Y. 2016. The complete mitochondria genome of *Calliphora vomitoria* (Diptera: Calliphoridae). Mitochondrial DNA Part B. 1(1):378–379. 33473489 10.1080/23802359.2016.1159930 PMC7799468

[CIT0015] Sarmiento ME, Chin KL, Lau NS, Aziah I, Ismail N, Norazmi MN, Acosta A, Yaacob NS. 2021. Comparative transcriptome profiling of horseshoe crab *Tachypleus gigas* hemocytes in response to lipopolysaccharides. Fish Shellfish Immunol. 117:148–156. 34358702 10.1016/j.fsi.2021.08.001

[CIT0016] Simpson SD, Ramsdell JS, Watson WH, III, Chabot CC. 2017. The draft genome and transcriptome of the Atlantic horseshoe crab, *Limulus polyphemus*. Int J Genomics. 2017:1–14. 10.1155/2017/7636513 PMC5317147 28265565

[CIT0017] Staegemann MW. 2016. [Drivers of bat fly diversity and prevalence of six *Rhinolophus* bat species in southern Africa] [doctoral dissertation]. Westville: University of KwaZulu-Natal.

[CIT0018] Theodor O, Moscona A. 1954. On the bat parasites in Palestine I. Nycteribiidae, Streblidae, Hemiptera, Siphonaptera. Parasitology. 44(1-2):157–245. 13166381 10.1017/s0031182000018862

[CIT0019] Theodor O. 1967. An illustrated catalogue of the Rothschild collection of Nycteribiidae (Diptera) in the British Museum (Natural History); with keys and short descriptions for the identification of subfamilies, genera, species and subspecies. London: British Museum (Natural History).

[CIT0020] Tillich M, Lehwark P, Pellizzer T, Ulbricht-Jones ES, Fischer A, Bock R, Greiner S. 2017. GeSeq–versatile and accurate annotation of organelle genomes. Nucleic Acids Res. 45(W1):W6–W11. 28486635 10.1093/nar/gkx391 PMC5570176

[CIT0021] Trevisan B, Alcantara DM, Machado DJ, Marques FP, Lahr DJ. 2019. Genome skimming is a low-cost and robust strategy to assemble complete mitochondrial genomes from ethanol preserved specimens in biodiversity studies. PeerJ. 7:e7543. 31565556 10.7717/peerj.7543 PMC6746217

[CIT0022] Trifinopoulos J, Nguyen LT, von Haeseler A, Minh BQ. 2016. W-IQ-TREE: a fast online phylogenetic tool for maximum likelihood analysis. Nucleic Acids Res. 44(W1):W232–W235. 27084950 10.1093/nar/gkw256 PMC4987875

[CIT0023] Zhao Z, Su TJ, Chesters D, Wang SD, Ho SYW, Zhu CD, Chen XL, Zhang CT. 2013. The mitochondrial genome of *Elodia flavipalpis* Aldrich (Diptera: Tachinidae) and the evolutionary timescale of tachinid flies. PLOS One. 8(4):e61814. 23626734 10.1371/journal.pone.0061814 PMC3634017

[CIT0024] Zhou Q, Ding S, Li X, Zhang T, Yang D. 2017. Complete mitochondrial genome of A*llognosta vagans* (Diptera, Stratiomyidae). Mitochondrial DNA Part B. 2(2):461–462. 33473862 10.1080/23802359.2017.1357450 PMC7800880

